# Tuberculous Granuloma Formation Is Enhanced by a *Mycobacterium* Virulence Determinant

**DOI:** 10.1371/journal.pbio.0020367

**Published:** 2004-10-26

**Authors:** Hannah E Volkman, Hilary Clay, Dana Beery, Jennifer C. W Chang, David R Sherman, Lalita Ramakrishnan

**Affiliations:** **1**Molecular and Cellular Biology Graduate Program, University of WashingtonSeattle, WashingtonUnited States of America; **2**Department of Microbiology, University of WashingtonSeattle, WashingtonUnited States of America; **3**Department of Pathobiology, University of WashingtonSeattle, WashingtonUnited States of America; **4**Department of Immunology, University of WashingtonSeattle, WashingtonUnited States of America; **5**Department of Medicine, University of WashingtonSeattle, WashingtonUnited States of America

## Abstract

Granulomas are organized host immune structures composed of tightly interposed macrophages and other cells that form in response to a variety of persistent stimuli, both infectious and noninfectious. The tuberculous granuloma is essential for host containment of mycobacterial infection, although it does not always eradicate it. Therefore, it is considered a host-beneficial, if incompletely efficacious, immune response. The *Mycobacterium* RD1 locus encodes a specialized secretion system that promotes mycobacterial virulence by an unknown mechanism. Using transparent zebrafish embryos to monitor the infection process in real time, we found that RD1-deficient bacteria fail to elicit efficient granuloma formation despite their ability to grow inside of infected macrophages. We showed that macrophages infected with virulent mycobacteria produce an RD1-dependent signal that directs macrophages to aggregate into granulomas. This *Mycobacterium*-induced macrophage aggregation in turn is tightly linked to intercellular bacterial dissemination and increased bacterial numbers. Thus, mycobacteria co-opt host granulomas for their virulence.

## Abstract 2

A zebrafish model of mycobacterium infection demonstrates that formation granulomas - immune structures composed of macrophages - requires a bacterial gene, indicating that mycobacteria exploit granuloma formation for infection

## Introduction

Infection with pathogenic mycobacteria is thought to proceed through a series of defined steps. Mononuclear cells present at or recruited to sites of infection phagocytose bacteria and migrate deeper into tissues. Then additional macrophages and other immune cells are recruited to form complex, tightly aggregated structures called granulomas (Adams 1976; Dannenberg 1993; Teitelbaum et al. 1999; Geijtenbeek et al. 2003; Peters and Ernst 2003; Tailleux et al. 2003; Cosma et al. 2004). Granuloma macrophages subsequently undergo differentiation into epithelioid cells, so called owing to their closely apposed cellular membranes (Adams 1976; Dannenberg 1993; Cosma et al. 2004). Production and maintenance of granulomas is essential to the control of tuberculosis in murine models and humans (Kaufmann 2000; Flynn and Chan 2001; Lawn et al. 2002). However, despite residence at the site of a robust focal immune response, the bacilli within granulomas are not always eradicated (Cosma et al. 2003; Cosma et al. 2004). The relative contributions of host and pathogen determinants to the migration and aggregation of macrophages and the formation and maintenance of granulomas are not understood.

While considerable progress has been made in identifying *Mycobacterium* virulence determinants (Glickman and Jacobs 2001; Cosma et al. 2003; Smith 2003), our overall understanding of mycobacterial pathogenesis remains rudimentary. Virulence determinants are generally studied by examining mutant bacterial strains in cultured macrophage monolayers or by static assessment of bacterial numbers and tissue pathology at different time points during in vivo infection. During in vivo infection, spatially separated individual mononuclear cells are infected, migrate into tissues, and serve as a nidus for cellular aggregation (Teitelbaum et al. 1999; Davis et al. 2002; Geijtenbeek et al. 2003; Tailleux et al. 2003). The steps and dynamics of bacterially mediated host-cell interactions that impact the outcome of infection through production of chemokines, cytokines, adhesion molecules, and their receptors cannot be elucidated by tissue culture studies and static assessments in vivo. To address these issues, we utilize a novel model of *Mycobacterium* infection in zebrafish. Zebrafish embryos and larvae (henceforth we will refer to both stages as embryos) are naturally susceptible to infection by *Mycobacterium marinum,* and we have previously shown that their optical transparency may be used to monitor the cellular dynamics of infection in real time (Davis et al. 2002). Using differential interference contrast (DIC) video and fluorescence microscopy, we have monitored macrophage chemotaxis to *M. marinum,* its phagocytosis, transit of infected macrophages into tissues, and the recruitment of additional macrophages to initiate granulomas that have the pathological hallmarks and bacterial gene expression profile characteristic of tuberculous granulomas in adult animals.

In this study, we use the zebrafish infection model to probe the cellular mechanisms of virulence of the *Mycobacterium* RD1 locus, an approximately 10-kb region missing from all attenuated bacille Calmette-Guérin (BCG) vaccine strains but present in virulent M. tuberculosis isolates (Mahairas et al. 1996; Behr and Small 1999). The RD1 locus encodes a specialized secretion system for the putative virulence effector proteins ESAT-6 and CFP-10, also located within the locus (Tekaia et al. 1999; Pallen 2002; Hsu et al. 2003; Pym et al. 2003; Stanley et al. 2003; Guinn et al. 2004). The M. tuberculosis RD1 deletion mutant has a growth defect in the mouse model of tuberculosis (Lewis et al. 2003), and studies using cultured macrophages and other in vitro systems have identified complex phenotypes that may account for its in vivo attenuation (Hsu et al. 2003; Stanley et al. 2003; Guinn et al. 2004). In vitro assays have suggested that RD1 may contribute to mycobacterial cytotoxicity to macrophages and epithelial cells, thus enabling bacterial spread between cells or transit across epithelial barriers (Hsu et al. 2003; Guinn et al. 2004). Others have proposed that the RD1 region mediates dampening of host innate immune responses in macrophages (Stanley et al. 2003). It is unclear how each of these individual in vitro phenotypes contributes to the complex sequence of events that ultimately lead to bacterial persistence in granulomas. We used the zebrafish-M. marinum infection model to elucidate the precise steps at which infection with wild-type (WT) and RD1 mutant bacteria differ. Our data suggest that the RD1 locus independently mediates macrophage aggregation and intercellular bacterial spread via host cell death within aggregates. These steps are associated with increased bacterial numbers and enhanced virulence, lending support to the idea that mycobacteria actually promote and exploit granuloma formation for the establishment of infection.

## Results

### The M. marinum RD1 Mutant Is Attenuated for Growth in Cultured Macrophages and Adult Frogs

**Figure 1:**
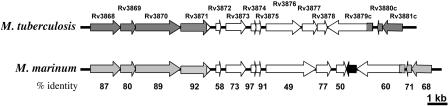
The RD1 Regions in M. tuberculosis and M. marinum Are Homologous and Syntenic The white arrows represent the RD1 region deleted from M. tuberculosis. The black arrow represents a predicted open reading frame not present in *M. tuberculosis.* Rv3874 and Rv3875 are also known as *cfp-10* and *esat-6,* respectively. Numbers represent the percent amino acid identities between the corresponding proteins of the two organisms.

**Figure 2:**
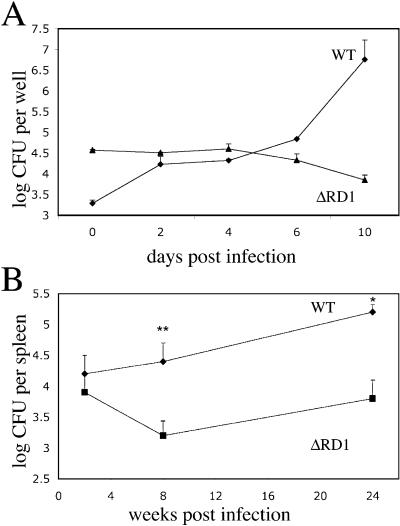
M. marinum ΔRD1 Is Attenuated In Vitro and In Vivo (A) Growth of M. marinum WT and ΔRD1 in J774 cells. Each time point represents the average of triplicate values. Error bars are ± standard error of the mean (SEM). (B) WT and ΔRD1 bacterial numbers in frog spleens. Each time point represents the average colony counts from 3–5 frogs. Error bars are ± SEM (* *p* ≤ 0.05, ** *p* = 0.016, unpaired Student's *t*-test). Infecting doses were 5.8 × 10^5^ CFU for WT and 1.2 × 10^6^ CFU for ΔRD1.

The genes in the M. marinum RD1 region are homologous to those in M. tuberculosis (for instance, their ESAT-6 and CFP-10 proteins are 97% and 91% identical, respectively) and the regions in the two organisms are syntenic (http://www.sanger.ac.uk/Projects/M_marinum/; Figure 1). We derived an M. marinum RD1-deficient mutant with essentially the same deletion as the M. tuberculosis RD1 mutant described previously (Figure 1) (Lewis et al. 2003). Like the M. tuberculosis RD1 mutant (Lewis et al. 2003), the M. marinum mutant (referred to as ΔRD1) was attenuated for growth in mouse and human monocyte/macrophage cell lines (Figure 2A and unpublished data). ΔRD1 was also attenuated for growth in an adult leopard frog infection model (Figure 2B) in which WT M. marinum causes chronic granulomatous infection (Ramakrishnan et al. 1997). Specifically, significantly fewer ΔRD1 bacteria were recovered from spleens and livers of infected frogs at 2, 8, and 24 wk postinfection (Figure 2B and unpublished data). ΔRD1-infected frogs also had poorly formed macrophage aggregates at 8 wk postinfection, in contrast to the well-defined granulomas resulting from WT infection (unpublished data). Thus, by previously evaluated parameters, the RD1 region plays identical roles in the virulence of M. tuberculosis and M. marinum.

### M. marinum ΔRD1 Infection of Zebrafish Embryos Results in Reduced Macrophage Aggregation

**Figure 3:**
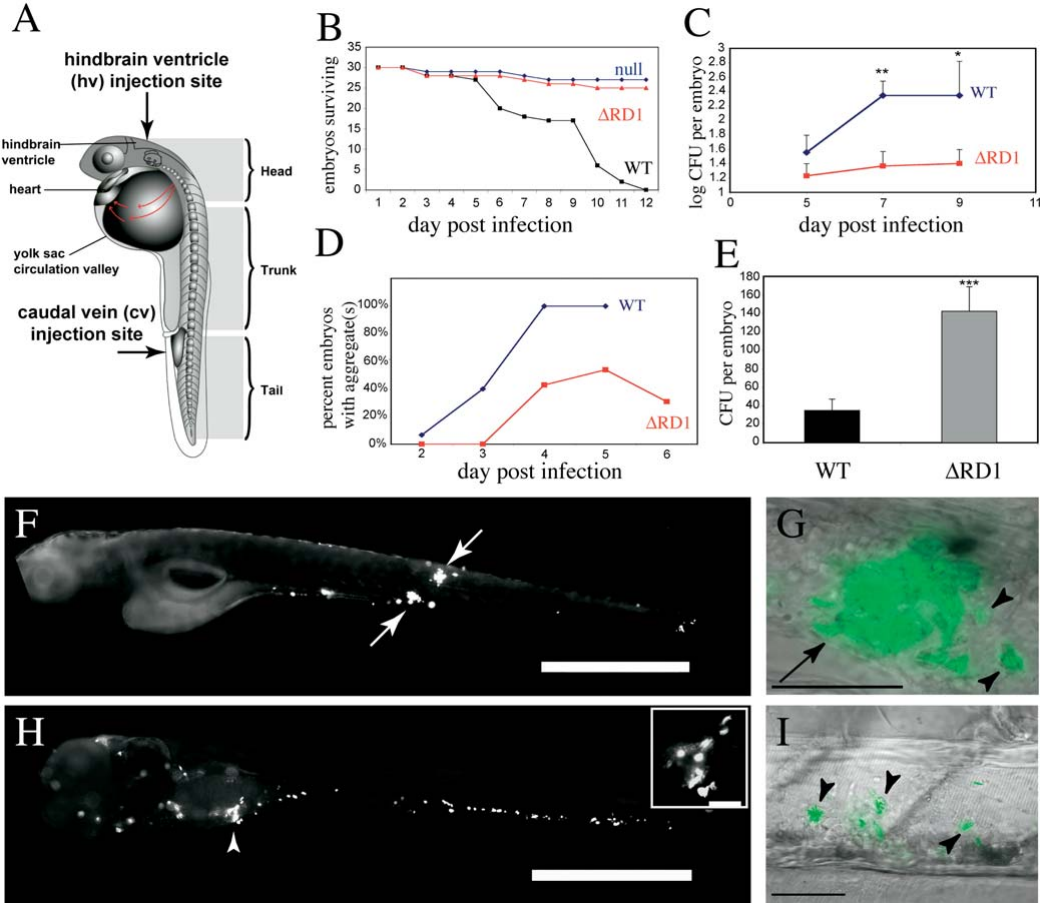
ΔRD1 Is Attenuated in Zebrafish Larvae (A) Diagram of the zebrafish embryo/larva. Arrows indicate the two injection sites used in this study. (B) Survival of embryos infected with ΔRD1 (410 CFU) or WT bacteria (250 CFU) and null-injected embryos. (C) Whole embryo bacterial counts of WT- and ΔRD1-infected embryos. Infecting doses: 32 CFU for WT, 36 CFU for ΔRD1. Error bars are ± SEM (** *p* = 0.0075 comparing 7-d postinfection WT to 7-d postinfection ΔRD1; * *p* = 0.05 comparing 9-d postinfection WT to 9-d postinfection ΔRD1, unpaired Student's *t*-test). (D) Time of aggregate formation, showing delayed aggregation in the ΔRD1-infected embryos (*n* = 13) as compared to WT-infected embryos (*n* = 15). Infecting doses: 131 CFU for WT, 301 CFU for ΔRD1. (E) Whole embryo bacterial counts of WT- and ΔRD1-infected embryos on day of aggregate formation. Infecting doses: 36 CFU for WT, 78 CFU for ΔRD1. Error bars are ± SEM (*** *p* = 0.0008, unpaired Student's *t*-test; ΔRD1 *n* = 28, WT *n* = 29). (F) Fluorescent image of WT-infected embryo at 6 d postinfection with two aggregates (arrows). Scale bar, 200 μm. (G) WT-infected embryos with higher magnification overlay of fluorescent and DIC images showing an aggregate (arrow) with individual infected macrophages that are migrating toward aggregate (arrowheads). Scale bar, 50 μm. (H) Fluorescent image of ΔRD1-infected embryo at 6 d postinfection that has not formed any aggregates. Note the numerous infected macrophages throughout the head, body, and tail. Arrowhead and close-up insert (scale bar, 50 μm) show infected macrophages close to each other, but not aggregating. Scale bar, 200 μm. (I) ΔRD1-infected embryo under higher-magnification overlay of DIC and fluorescent images showing three individual infected macrophages (arrowheads). Scale bar, 50 μm.

We injected fluorescent WT or ΔRD1 bacteria via the caudal vein directly into the bloodstream of 30 h postfertilization embryos (Figure 3A), which were then monitored for survival and bacterial load (Figure 3B and 3C; Materials and Methods) (Davis et al. 2002). In contrast to WT bacteria, ΔRD1 failed to kill the embryos during the 12 d monitoring period (Figure 3B). Consistent with this difference in mortality, ΔRD1 bacterial growth was attenuated as compared to WT bacteria in the embryos (Figure 3C).

**Figure 4:**
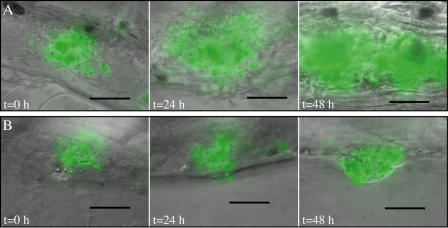
Progression of Aggregates a WT Aggregate (A), and a ΔRD1 Aggregate (B) (A) WT aggregates shown on the first day of aggregate formation (*t* = 0 h); 24 h after aggregate formation (*t* = 24 h); and 48 h after aggregate formation (*t* = 48 h). (B) ΔRD1 aggregates shown at the same time points as in (A). A 60× water lens was used for all photomicrographs except the image in (A) *t* = 48 h, which was taken with a 40× lens. Scale bar represents 50 μm.

To understand the cellular basis of ΔRD1 attenuation, we undertook real-time microscopic monitoring of the infection process with WT and mutant bacteria. WT infection of the embryos is characterized by the transit of infected macrophages into tissues where macrophages are recruited to form granulomas within 3–5 d postinfection (Figure 3D, 3F, and 3G) (Davis et al. 2002). In contrast, while ΔRD1 infected macrophages also migrated from the circulation to the tissues (Figure 3H), fewer, if any, aggregates formed, and the kinetics of their formation were delayed compared to the WT-infected cells (Figure 3D). Several highly infected individual macrophages were found scattered throughout the tissues, often close to each other (Figure 3H and 3I). This is in sharp contrast to the case of WT infection, in which infected macrophages are nearly always found in aggregates (Davis et al. 2002). Aggregates that formed in ΔRD1-infected embryos were more transient than those in WT-infected embryos, often dissociating into individual infected macrophages (Figure 3D and unpublished data). Also, the ΔRD1 aggregates remained small in contrast to WT aggregates, which often increased dramatically in size (Figure 4, compare images in [A] to those in [B]). This finding suggests that RD1 is required not only to initiate aggregate formation but for an ongoing recruitment of macrophages into the aggregate.

Since M. marinum ΔRD1 is attenuated for growth in the embryos (see Figure 3C), we considered the possibility that this mutant strain did not replicate enough to reach the threshold bacterial numbers that might be required to stimulate host pathways for macrophage aggregation. In that case, the inability of ΔRD1 to induce macrophage aggregation would be a simple consequence of its primary replication defect in the embryos. This scenario would predict that the number of bacteria required for aggregate formation would be similar in WT and ΔRD1 infection. To investigate this possibility, we infected embryos with similar numbers of the two bacterial strains and examined them daily for aggregate formation. On the day each embryo developed an aggregate(s), it was lysed and bacterial colony-forming units (CFU) determined. The bacterial load at which macrophage aggregation first occurred was over 4-fold higher in ΔRD1- than WT-infected embryos (see Figure 3E). These results show that ΔRD1 infection is associated with a primary aggregation defect that is not a consequence of its decreased replication in macrophages.

### The RD1 Locus Specifically Mediates Macrophage Aggregation

**Figure 5:**
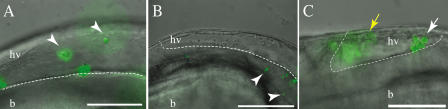
Normal Macrophage Chemotaxis to Initial Sites of ΔRD1 Infection Overlay of DIC and fluorescent images showing the hindbrain ventricle (hv) of infected embryos. The hindbrain ventricle/brain (hv/b) boundary indicated by a white dashed line. (A) ΔRD1-infected embryo 4 h postinfection with individual infected macrophages marked by arrowheads. (B) ΔRD1-infected embryo 5 h postinfection in which individual infected macrophages (arrowheads) have migrated from the hindbrain ventricle and into the brain. (C) WT-infected embryo 24 h postinfection with macrophages beginning to aggregate (white arrow) in the hindbrain ventricle. A second out-of-focus aggregate is to the left (yellow arrow). Scale bar, 100 μm.

Macrophages are rapidly recruited to the site of WT M. marinum infection, where they phagocytose the bacteria, migrate to the tissues, and form aggregates (Davis et al. 2002). Since macrophage migration and aggregation are likely mediated by as yet ill-defined chemotactic networks, we asked if the RD1 locus also affected other chemotactic macrophage functions. Macrophage recruitment is most stringently assessed by injecting bacteria into the hindbrain ventricle, an isolated cavity devoid of macrophages in the absence of bacteria (see Figure 3A) (Herbomel et al. 1999; Davis et al. 2002). Similar numbers of macrophages migrated to the hindbrain ventricle in response to the injection of WT and ΔRD1 bacteria at 4 h postinfection, and most of the bacteria had been phagocytosed in both cases (Figure 5A; unpublished data). Therefore, the RD1 locus does not affect macrophage chemotaxis to the bacteria or phagocytic capabilities.

The abundance of ΔRD1-infected macrophages in tissues following bloodstream infection (see Figure 3H and 3I) suggested that the RD1 locus is not required for tissue migration following infection. However, tissue migration can also be examined more stringently following the ventricle injection assay (Davis et al. 2002). 5 h after infection of the ventricle, many of the ΔRD1-infected macrophages had entered the brain tissue (Figure 5B). By 24 h, most of the macrophages were widely disseminated throughout the tissues (unpublished data). Indeed, the lack of aggregation by RD1-infected macrophages led to their enhanced tissue dissemination compared to WT-infected macrophages, which had often formed aggregates within the ventricle itself by 24 h postinfection (Figure 5C). Likely as a result, the WT-infected macrophages were slower to migrate out of the ventricle than ΔRD1-infected macrophages. Even when they did migrate out of the ventricle, they often formed aggregates in the adjacent brain tissue and did not disseminate into the trunk and tail as rapidly as did the ΔRD1-infected macrophages. In summary, the ventricle infections showed that the RD1 locus is not required for macrophage chemotaxis to the site of infection, bacterial phagocytosis, or tissue migration of infected macrophages. Furthermore, this assay highlighted the difference in the aggregation of WT- and ΔRD1-infected macrophages from very early in infection.

### ΔRD1-Infected Macrophages Can Receive, but Not Send, Signals That Promote Aggregation

**Figure 6:**
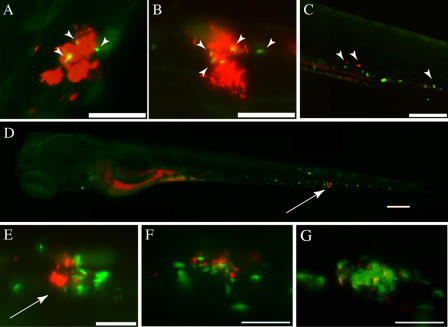
Superinfection with WT Bacteria Rescues ΔRD1 Aggregation Defect (A and B) Embryos with aggregates at 3d postinfection with 85 CFU red-fluorescent WT bacteria are shown 4 h after superinfection with green-fluorescent strains of either ΔRD1 (134 CFU) (A) or WT (169 CFU) (B) bacteria. Superinfecting strains were injected at sites distant from the aggregates, and pictures were taken outside of injection regions. Arrowheads indicate macrophages infected with superinfecting strain. Scale bar, 100 μm. (C) Embryo infected with 171 CFU green-fluorescent ΔRD1 for 4 d shown 4 h post-superinfection with 364 CFU of red-fluorescent ΔRD1. Arrowheads point to macrophages infected with each of the bacterial strains. Scale bar, 200 μm. (D) Embryo infected with 171 CFU green-fluorescent ΔRD1 for 4 d shown 4 h after superinfection with 363 CFU of red-fluorescent WT bacteria. Arrow points to macrophage aggregate. Scale bar, 200 μm. (E) Higher magnification image of aggregate (arrow) in (D) showing green fluorescent ΔRD1 and red fluorescent WT bacteria. Arrowhead points to WT-infected macrophage outside the aggregate. Scale bar, 50 μm. (F and G) Embryo infected with green fluorescent ΔRD1, superinfected with red fluorescent WT (as in D and E) shown at 24 h post-superinfection (F), and the same aggregate at 48 h post-secondary infection (G). Scale bars, 50 μm. All panels are fluorescent images.

The aggregation defect of ΔRD1-infected macrophages suggests that they lack the capacity to either produce or receive signals that mediate aggregation of macrophages during WT infection. To begin to dissect the nature of the missing signal(s), we infected embryos with red-fluorescent WT bacteria and allowed aggregates to form. These embryos were then superinfected with green-fluorescent ΔRD1 or WT bacteria (Figure 6). Both superinfecting strains were phagocytosed by individual macrophages, which migrated in similar numbers to preexisting aggregates within 4 h (Figure 6A and 6B). These data indicate that ΔRD1-infected macrophages can receive signals produced by WT-infected macrophages and migrate rapidly toward aggregates.

**Figure 7:**
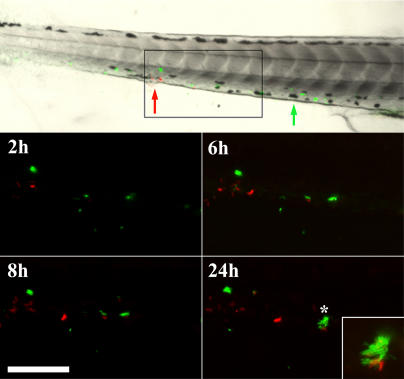
Superinfection with WT Bacteria Rescues ΔRD1 Aggregation Defect over Time Embryos were injected with fluorescent ΔRD1 (green) at 1 d postfertilization. 3 d post-primary infection, embryos were injected with fluorescent WT (red) and followed for 24 h post-secondary infection. Approximate injection sites are shown with green and red arrows for ΔRD1 and WT bacteria, respectively. Box in top panel indicates the magnified field in fluorescent images. Inset panel at 24-h time point is a magnified image of the starred aggregate. Scale bar, 125 μm.

In a reciprocal experiment, embryos were first infected with green-fluorescent ΔRD1 bacteria, and after 4 d, when there were abundant individual infected macrophages (but no aggregates) in the tissues, the embryos were superinfected with either WT or ΔRD1 red-fluorescent bacteria. As expected, both superinfecting strains were rapidly phagocytosed by uninfected macrophages. ΔRD1 superinfection did not cause aggregate formation, and individual macrophages carrying both the original or superinfecting ΔRD1 were scattered throughout the tissues (Figure 6C). In contrast, WT-infected macrophages induced the aggregation of preexisting ΔRD1-infected macrophages as early as 4 h after superinfection (Figure 6D, 6E, and 7). These newly formed aggregates were often composed mostly of ΔRD1-infected macrophages with only a few WT-infected macrophages in them (Figure 7). All aggregates had at least some WT-infected macrophages. Furthermore, ΔRD1/WT aggregates that formed developed normally, increasing in size and recruiting both ΔRD1- and WT-infected macrophages (Figure 6F and G). Thus, this experiment confirmed that ΔRD1-infected macrophages receive but do not send aggregation signals and have no intrinsic chemotactic defects. Furthermore, it appears that WT-infected macrophages are required to serve as a nidus for each aggregate, suggesting that they create a chemotactic gradient that recruits macrophages.

### Macrophage Aggregation Is Tightly Linked to Intercellular Bacterial Spread

Having demonstrated that ΔRD1 infection results in both reduced aggregation and lower bacterial numbers, we next pursued experiments to determine the relationship between these two phenotypes. In contrast to the notion that a primary reduction in bacterial numbers obviated the need for aggregation (see Figure 3E), we found that more ΔRD1 than WT bacteria were required for aggregates to form. Therefore, we sought to determine if, conversely, the aggregation defect resulted in reduced bacterial numbers.

**Figure 8:**
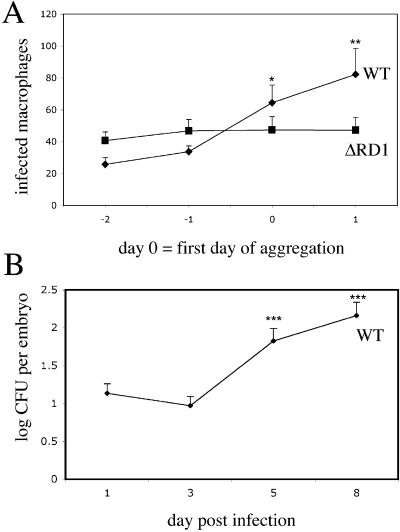
Macrophage Aggregation Correlates with Bacterial Dissemination during WT Infection (A) Enumeration of infected macrophages in embryos by fluorescent and DIC microscopy after infection with green-fluorescent bacteria. Infecting doses: 151 CFU for WT, 301 CFU for ΔRD1. Time points are in reference to day of aggregate formation, which is set at 0. 15 WT infected embryos and 13 ΔRD1 embryos were monitored. The graph represents all 15 WT embryos, but only the 7/13 ΔRD1 infected embryos that formed aggregates over the course of the experiment. Error bars are ± SEM. (*p = 0.0136 comparing WT day 0 and WT day –2; ** *p* = 0.0053 comparing WT day 1 and WT day –2, unpaired Student's *t*-test). (B) Whole embryo bacterial counts following WT infection (*** *p* ≤ 0.0003, 5 d postinfection and 8 d postinfection, respectively, compared to 3 d postinfection, unpaired Student's *t*-test).

One way that aggregation could impact bacterial numbers is by facilitating the spread of bacteria to uninfected macrophages that are recruited to the aggregates. If so, then aggregate formation should correlate with a dramatic increase in the number of infected macrophages and bacterial burdens. To test this hypothesis, we assessed the number of infected macrophages and bacterial numbers in relation to the time of aggregate formation (Figure 8). We enumerated daily by microscopy the number of infected macrophages during the course of infection starting at 1 d postinjection of bacteria and continuing up to 2 d after aggregates formed (Figure 8A). We counted as day 0 the first day of aggregation. During WT infection, the number of infected macrophages did not change significantly until aggregates formed (Figure 8A). However, upon aggregation, the number of infected macrophages increased dramatically (Figure 8A). Similarly, the number of viable bacteria also did not increase until after aggregation occurred 3–5 d postinfection (Figure 8B). Taken together, these data suggest that during WT infection, macrophage aggregation promotes intercellular bacterial spread and an increased bacterial burden.

In the case of ΔRD1 infection, macrophage aggregation did not result in an increase in the number of infected macrophages (Figure 8A). This difference could be solely due to the ongoing defect in macrophage recruitment by the ΔRD1-containing aggregates. It could also involve additional pathways that result in decreased bacterial spread to uninfected macrophages in the aggregates. In either case, this result suggests that while aggregation is required for intercellular bacterial spread, it is not sufficient. Additional RD1-mediated events must occur to facilitate spread after aggregation.

### WT Aggregates Have More Cell Death Than ΔRD1 Aggregates

**Figure 9:**
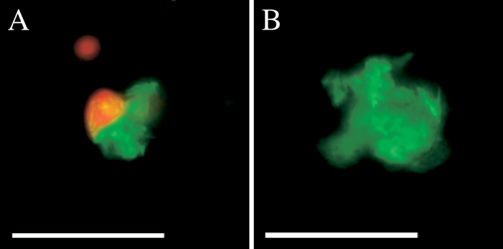
WT Aggregates Are More Likely to Have TUNEL-Positive Cells Than ΔRD1 Aggregates Representative fluorescent images of aggregates following TUNEL staining of 6-d postfertilization embryos infected with 71 green-fluorescent WT (A), or 474 green-fluorescent ΔRD1 (B) bacteria. TUNEL staining is imaged with red fluorescence, and colocalization with green-fluorescent bacteria appears yellow. Scale bar, 100 μm.

Having established that RD1 is involved in macrophage recruitment to aggregates, we sought to determine if it also affects intercellular bacterial spread by additional means. The M. tuberculosis RD1 locus is thought to promote intercellular bacterial spread in confluent cultured macrophage monolayers by promoting death of infected cells and subsequent phagocytosis of the released bacteria by surrounding cells (Guinn et al. 2004). Therefore, we hypothesized that RD1 might operate similarly in the embryo aggregates to mediate bacterial spread by facilitating cell death. For this assessment, we achieved comparably sized aggregates with the two strains by infecting embryos with 6.6-fold more ΔRD1 than WT bacteria, and performed terminal deoxynucleotidyl transferase–mediated deoxyuridine triphosphate nick end labeling (TUNEL) staining on whole infected embryos to visualize dead and dying cells. The TUNEL reaction labels double-stranded DNA breaks that can occur during apoptosis and certain forms of necrosis (Gavrieli et al. 1992). WT infected embryos had more aggregates with TUNEL-positive cells (15 of 23) than did ΔRD1-infected embryos (6 of 22) (Figure 9). Contingency table analysis revealed that WT aggregates were 2.3 times more likely to contain TUNEL-positive cells than ΔRD1 aggregates (*p* = 0.017, Fisher's exact *t*-test). The TUNEL-positive cells were often M. marinum-infected (Figure 9A). Furthermore, TUNEL-positive infected cells were found almost exclusively within aggregates. Taken together, our data suggest that the RD1 locus first mediates macrophage aggregation and subsequently promotes cell death within the aggregates.

### The RD1 Locus Continues to Mediate Granuloma Formation During Long-Term Infection

**Figure 10:**
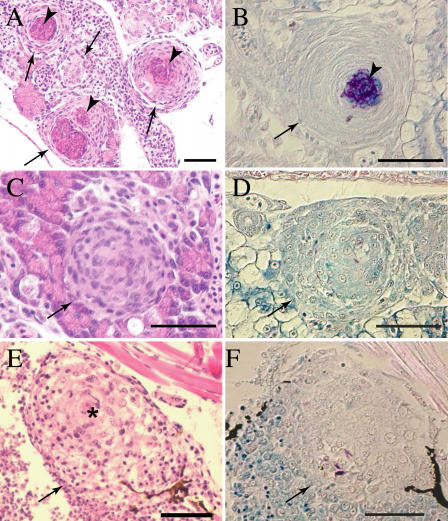
ΔRD1 Infection Is Associated with Persistent Defects in Granuloma Organization Tissue histology of 32-d postfertilization fish infected with either 21 WT (A–D) or 9 ΔRD1 (E–F) bacteria (doses were not significantly different *p* = 0.15) at 1 d postfertilization. Arrows indicate granulomas and loose aggregates, arrowheads indicate caseum. Hematoxylin and eosin staining are shown in (A), (C), and (E), and modified acid-fast staining is shown in (B), (D), and (F). (A) Organized caseating WT granulomas (arrow) with central caseum (arrowhead). (B) WT granuloma showing mycobacteria predominantly in caseum with a few within epithelioid cells. (C) Noncaseating but highly organized WT M. marinum-induced granulomas showing the expected few bacteria within cells in (D). (E) Large, loose, and poorly organized macrophage aggregate of ΔRD1-infected fish with evidence of epithelioid transformation only in the center (denoted by *). (F) A few mycobacteria in the ΔRD1 aggregates. Scale bar, 100 μm. Images in (A–D) were taken with a 40× lens, whereas those in (E) and (F) were taken with a 20× lens.

We have previously used the zebrafish embryo model to demonstrate that granulomas can be initiated solely by interactions of mycobacteria with innate immunity (Davis et al. 2002). However, their maturation and an enhancement in their mycobacteriocidal potential likely requires the participation of adaptive immunity (Flynn and Chan 2001; Davis et al. 2002). Thus, the zebrafish embryo model has been useful to separate the effects of innate and adaptive immunity on granuloma formation (Davis et al. 2002). Since both the M. tuberculosis and M. marinum RD1 mutants exhibit a sustained attenuation during chronic infection of adult animals (see Figure 2B) (Lewis et al. 2003), we wished to determine if the early aggregation defect we had discovered using the zebrafish embryo model impacts granuloma formation and maturation later in infection. We infected embryos with low doses of either WT or ΔRD1 bacteria and confirmed infection microscopically at 6 d postfertilization. Using these low infection doses, we could raise a very few WT-infected embryos to adulthood. In contrast, the mortality of the ΔRD1-infected embryos was no different from that of uninfected embryos during a 32 d observation period (see Figure 3B) (unpublished data). At 32 d, the fish were assessed by tissue histopathology. Mycobacteria were identified within the granulomas in all of the surviving fish, showing that they were chronically infected (Figure 10). WT-infected fish had highly organized granulomas, both caseating and noncaseating in roughly equal proportions (Figure 10A and 10C), with bacteria located predominantly in the caseum (Figure 10B and 10D). These granulomas appeared identical to granulomas resulting from infection of adult zebrafish (Cosma et al. 2004). In contrast, ΔRD1-infected fish had only a few granulomas (Figure 10E and 10F), all of which were noncaseating and markedly different even from WT noncaseating granulomas (Figure 10C and 10E). The WT-induced granulomas were compact and were composed of tightly packed cells that displayed the indistinct cytoplasmic borders and abundant eosinophilic cytoplasm characteristic of epithelioid cells (Figure 10C) (Adams 1976; Bouley et al. 2001). In contrast, the ΔRD1-induced aggregates had more loosely aggregated cells, with evidence of epithelioid transformation only in the centers of some (Figure 10E).

In summary, the ΔRD1-induced macrophage aggregates in infected embryos raised to adulthood had the same lack of organization as the lesions resulting from infection of adult animals (unpublished data) (Sherman et al. 2004). These studies link our early real-time observations of phenotypes in the context of innate immunity alone to these seen later in infection. It appears that while some macrophage aggregation does occur in the absence of RD1, this locus continues to mediate aspects of macrophage chemotaxis and/or differentiation that contribute to granuloma architecture even as the infection becomes chronic.

## Discussion

We used the zebrafish-M. marinum infection model to identify the steps at which the *Mycobacterium* RD1 virulence locus impacts the infection process. We found that two steps are independently affected: macrophage aggregation into granulomas and intercellular spread therein. Promotion of cell death within these aggregates appears to be at least one of the means by which RD1 affects intercellular spread. By comparing WT and ΔRD1 infection in real time, we have uncovered the promotion of granuloma formation as a mechanism of *Mycobacterium* virulence.

The RD1 locus has been the recent subject of attention following its discovery as a major factor in the attenuation of BCG (Pym et al. 2002; Lewis et al. 2003) and the potential of an M. tuberculosis RD1-defective strain as a candidate vaccine for tuberculosis (Hsu et al. 2003; Pym et al. 2003). Subsequent studies suggested that it encodes a novel specialized secretion system for specific virulence effectors (Hsu et al. 2003; Pym et al. 2003; Stanley et al. 2003; Guinn et al. 2004). Deletion of the RD1 locus in M. tuberculosis and M. marinum results in reduced bacterial numbers during infection of cultured macrophages and adult animals (Hsu et al. 2003; Lewis et al. 2003; Stanley et al. 2003; Guinn et al. 2004; this study). Different in vitro studies have implicated the RD1 locus and its effectors ESAT-6 and CFP-10 in a variety of functions including lysis of cultured macrophage in confluent monolayers to promote intercellular spread (Guinn et al. 2004), disruption of artificial membranes (taken as a surrogate for lysis of host epithelial cell layers) (Hsu et al. 2003), and dampening of macrophage proinflammatory responses (Stanley et al. 2003). Whether these in vitro activities operate in vivo and how they impact virulence are not known. As is the case with virtually all *Mycobacterium* virulence determinants, the precise steps at which RD1 impacts virulence have not been elucidated.

The *Mycobacterium* infection model used here allows monitoring of the earliest individual stages of infection in real-time (Davis et al. 2002). Using this model, we were able to confirm that RD1 promotes macrophage death in vivo. Additionally, we have shown that the RD1 locus affects an unanticipated earlier step in pathogenesis, macrophage aggregation. Aggregation of infected cells is independent of bacterial replication within individual macrophages and distinct from other macrophage functions such as their chemotaxis to the bacteria and migration back to deeper tissues.

We speculate that RD1 mediates aggregation via its secreted effectors, which presumably interact with components of host macrophage signaling pathways to modulate macrophage aggregation. Some combination of chemokines, cytokines, and adhesion molecules is likely to be affected. Our superinfection experiments suggest a model by which RD1 impacts cellular signaling and aggregation. Because the ΔRD1-induced aggregates that form upon superinfection with WT bacteria always have at least one WT-infected macrophage, the RD1-induced signal likely diffuses from the infected macrophage to attract other macrophages to it to form aggregates. As ΔRD1-infected macrophages can receive but not send signals for aggregation, RD1 is likely required to induce expression of a chemotactic molecule but not its receptor.

The formation of the tuberculous granuloma requires a complex cascade of interrelated signals that mediate cell recruitment, adhesion, and differentiation. In our model, ΔRD1 infection results in alterations of all three processes. The initial lack of aggregation suggests a specific defect in the ability of ΔRD1-infected macrophages to recruit additional macrophages to form aggregates. Our real-time monitoring showed that ΔRD1-infected macrophages fail to aggregate even when they are in close proximity, suggesting that the primary defect in ΔRD1-infected macrophages is in the ability to send chemotactic signals for aggregation. On the other hand, our finding that ΔRD1-infected macrophage aggregates are more transient than WT ones may implicate both chemotactic and adhesion defects. This idea is further supported by the finding that the ΔRD1-induced lesions in the adult fish are composed of loosely aggregated macrophages with little epithelioid differentiation. However, a primary defect in the ability of ΔRD1-infected macrophages to send chemotactic signals could affect subsequent expression of adhesion molecules (Peters and Ernst 2003). Therefore we propose that the *Mycobacterium* RD1 locus induces infected macrophages to send chemotactic signals for aggregation of macrophages, which in turn affect adhesion and other downstream events that result in granuloma formation. Granuloma formation is not completely blocked upon infection with a ΔRD1 strain, as has been shown previously in the murine model of M. tuberculosis infection and in patient studies of disseminated BCG infection (Emile et al. 1997; Sherman et al. 2004). However, our data indicate that RD1 influences early aggregation events that seem to extend into later stages of infection.

Our data further suggest a model in which bacterial dissemination is facilitated by recruitment into the aggregates of uninfected macrophages that provide new habitats for further bacterial growth. Some ways in which incoming macrophages become infected could include transfer of bacteria between macrophages along membranous tethers (Davis et al. 2002), actin-based motility of extravacuolar bacteria leading to intercellular transfer (Stamm et al. 2003), and release of bacteria from dying infected cells. These dead cells could either release bacteria for phagocytosis by neighboring cells or be engulfed in their entirety (Ramakrishnan and Falkow 1994; Davis et al. 2002). All of these modes of bacterial transfer are likely to be enhanced by the close juxtaposition of macrophages within aggregates. Our data are consistent with earlier reports suggesting that RD1 mediates bacterially induced toxicity to host cells (Hsu et al. 2003; Guinn et al. 2004). While TUNEL staining is not a conclusive indication of apoptosis, it is most often associated with programmed cell death. In vitro studies indicate that apoptosis leads to bacterial cell death; however, our experiments indicate a correlation between host cell death and bacterial dissemination (Fratazzi et al. 1999). Since we did not observe TUNEL-positive infected cells prior to aggregation or outside of the aggregates, we believe that aggregation precedes cell death. Mechanistically, it is possible that RD1 effectors modulate impinge upon distinct signaling pathways for cell aggregation and death. Alternatively, RD1 may impact a common molecule, such as tumor necrosis factor, that affects both processes (Locksley et al. 2001). Other modes of bacterial transfer may also contribute to bacterial dissemination, and these may or may not be mediated by RD1.

Our examination of early infection events in vivo may serve to identify relevant findings from the in vitro studies. For instance, our data do not support the model that RD1 contains a cytolysin for epithelial cell barriers that allows mycobacteria to penetrate directly into deeper tissues (Hsu et al. 2003). Rather, these findings corroborate previous work from our laboratory and others showing that systemic dissemination of mycobacteria is effected mainly by trafficking of infected host mononuclear cells (Teitelbaum et al. 1999; Davis et al. 2002; Geijtenbeek et al. 2003; Tailleux et al. 2003; Cosma et al. 2004). We show, furthermore, that RD1 is not required for this early event. Another in vitro study describes the dampening of several macrophage innate immune responses, including the cytokine tumor necrosis factor, by WT but not RD1-mutant M. tuberculosis (Stanley et al. 2003). While this may be true in vivo as well, our functional approach shows that there is not a global dampening of chemotactic and innate immune responses by WT mycobacteria. Rather there is an RD1-mediated enhancement of macrophage aggregation and death.

Ultimately, our studies reveal that *Mycobacterium* expresses specific virulence factors that enhance macrophage aggregation into granulomas, starting very early after infection. This effect correlates with bacterial dissemination and an increase in the bacterial burden. Granulomas are thought to be primarily protective host immune structures that provide a focused immune response to restrict mycobacteria. According to prevailing models, recruitment and activation of additional macrophages provide a concentrated source of immune effectors that thwart the bacteria. The specific differentiation of macrophages into epithelioid cells with tightly interdigitated intercellular membranes helps sequester the infection. While there is clear evidence that granulomas are necessary for protection, there is increasing evidence that they are incompletely effective (Flynn and Chan 2001; Cosma et al. 2003; Cosma et al. 2004). We have recently shown that superinfecting mycobacteria traffic rapidly into preestablished granulomas, yet can survive therein (Cosma et al. 2004). The present study showing that mycobacteria promote the formation of these structures to enhance their dissemination reveals an even greater complexity in the granuloma's role in the pathogenesis of tuberculosis.

## Materials and Methods

### 

#### Construction of M. marinum strains

To generate a M. marinum RD1 mutant, PCR fragments immediately upstream (1,004 bp) and downstream (1,296 bp) of the region to be deleted (see Figure 1) were amplified from genomic DNA and cloned into the plasmid pKO, to flank a kanamycin resistance determinant (Sherman et al. 2001). The resulting plasmid, pJC2, was used to generate a RD1 deletion mutation in M. marinum as described (Ramakrishnan et al. 2000; Lewis et al. 2003). Both WT and ΔRD1 strains were transformed with plasmids containing transcriptional fusions of genes encoding either red-fluorescent protein *(dsRed2)* or green-fluorescent protein *(gfp)* to a constitutive M. marinum promoter as described (Chan et al. 2002; Cosma et al. 2004).

#### Macrophage infection assays

J774 mouse macrophage-like cells and THP1 human macrophage-like cells were grown and prepared for infection as described (Chan et al. 2002; Guinn et al. 2004). Infection with M. marinum and determination of intracellular bacterial counts was done as described (Chan et al. 2002).

#### Frog infections

Frogs were injected intraperitoneally with *M. marinum,* and tissue bacterial counts were obtained as described (Ramakrishnan et al. 1997).

#### Zebrafish embryo infections

Zebrafish embryos were maintained and injected with M. marinum strains as described (Davis et al. 2002).

#### Microscopy of embryos

DIC and video microscopy were performed using a Nikon E600 (Nikon, Tokyo, Japan) equipped with 10×, 20×, and 40× magnifications, or a Nikon DIC 60× water “fluor” objective. Fluorescent as well as black and white images were collected with a Photometrics CoolSnap “cf” camera (Roper Scientific, Trenton, New Jersey, United States). Overlays of DIC and fluorescent images and video compilations were produced by using Metamorph software as described (Davis et al. 2002).

#### Determination of whole embryo bacterial counts

Individual embryos were placed in microcentrifuge tubes containing 100 μl of embryo medium containing 20 μg/ml kanamycin for 1 h at room temperature. This medium was removed by aspiration and replaced with 150 μl of 0.25% Trypsin-EDTA. After incubation for 6–8 h at room temperature, Triton X-100 was added to a 0.1% final concentration, and the tubes were sonicated for 10 min in an ultrasonicator (Bransonic Ultrasonic Cleaner 1510R-NT; Branson Ultrasonics, Danbury, Connecticut, United States). The entire sample from each tube was plated onto individual 7H11 solid media plates containing 20 μg/ml kanamycin.

#### TUNEL assay

5 d following infection, embryos were fixed in 4% paraformaldehyde in PBS overnight, dehydrated in methanol for a minimum of 24 h at 4 °C, rehydrated in PBS in a graded series of 5-min washes (in 75% methanol in PBS, 50% methanol in PBS, and 25% methanol in PBS), and washed four or five times in PBST (0.5% Tween 20 in PBS). Embryos were permeabilized using 10 μg/ml proteinase K in PBST for 30 min at 37 °C, postfixed in 4% paraformaldehyde in PBS for 20 min, washed five times for 5 min each in PBST, and twice for 5 min each in TTase Buffer (25 mM Tris-HCl [pH 6.6], 0.2M sodium cacodylate, 0.25 mg/ml BSA, and 0.2% Tween 20) plus 1 mM CoCl. Embryos were then incubated with TUNEL enzyme (#1767305; Roche, Basel, Switzerland) and TUNEL label mix (#1767291, Roche) according to the manufacturer's specifications. Primary antibody staining with sheep anti-fluorescein (#1426338 at 1/10,000; Roche) was done in Western blocking solution (#1921673, Roche) overnight at 4 °C. Secondary antibody staining with horseradish peroxidase-conjugated rabbit anti-sheep (#313035047 at 1/500; Jackson Immunoresearch, Bar Harbor, Maine, United States) was done for 2 h at RT. Detection was done with Tyramide Amplification Signal kit with AlexaFluor 555 (Molecular Probes #T30953; Molecular Probes, Eugene, Oregon, United States) according to the manufacturer's specifications.

#### Tissue histology of adult fish

Fish were fixed in Dietrich's fixative (30% ethanol, 10% formalin, and 2% glacial acetic acid in deionized water) and sectioning and staining were performed by Histo-Tec (Hayward, California, United States).

#### Statistics.

Statistics were calculated using GraphPad InStat version 3.05.

## Abbreviations

BCG: bacille Calmette-Guérin
CFU: colony-forming units
DIC: differential interference contrast
SEM: standard error of the mean
TUNEL: terminal deoxynucleotidyl transferase–mediated deoxyuridine triphosphate nick end labeling
WT: wild-type

## References

[pbio-0020367-Adams1] Adams DO (1976). The granulomatous inflammatory response. A review. Am J Pathol.

[pbio-0020367-Behr1] Behr MA, Small PM (1999). A historical and molecular phylogeny of BCG strains. Vaccine.

[pbio-0020367-Bouley1] Bouley DM, Ghori N, Mercer KL, Falkow S, Ramakrishnan L (2001). Dynamic nature of host-pathogen interactions in Mycobacterium marinum granulomas. Infect Immun.

[pbio-0020367-Chan1] Chan K, Knaak T, Satkamp L, Humbert O, Falkow S (2002). Complex pattern of Mycobacterium marinum gene expression during long-term granulomatous infection. Proc Natl Acad Sci U S A.

[pbio-0020367-Cosma1] Cosma CL, Sherman DR, Ramakrishnan L (2003). The secret lives of the pathogenic mycobacteria. Annu Rev Microbiol.

[pbio-0020367-Cosma2] Cosma CL, Humbert O, Ramakrishnan L (2004). Superinfecting mycobacteria home to established tuberculous granulomas. Nat Immunol.

[pbio-0020367-Dannenberg1] Dannenberg AM (1993). Immunopathogenesis of pulmonary tuberculosis. Hosp Pract.

[pbio-0020367-Davis1] Davis JM, Clay H, Lewis JL, Ghori N, Herbomel P (2002). Real-time visualization of *Mycobacterium*-macrophage interactions leading to initiation of granuloma formation in zebrafish embryos. Immunity.

[pbio-0020367-Emile1] Emile JF, Patey N, Altare F, Lamhamedi S, Jouanguy E (1997). Correlation of granuloma structure with clinical outcome defines two types of idiopathic disseminated BCG infection. J Pathol.

[pbio-0020367-Flynn1] Flynn JL, Chan J (2001). Immunology of tuberculosis. Annu Rev Immunol.

[pbio-0020367-Fratazzi1] Fratazzi C, Arbeit RD, Carini C, Balcewicz-Sablinska MK, Keane J (1999). Macrophage apoptosis in mycobacterial infections. J Leukoc Biol.

[pbio-0020367-Gavrieli1] Gavrieli Y, Sherman Y, Ben-Sasson SA (1992). Identification of programmed cell death in situ via specific labeling of nuclear DNA fragmentation. J Cell Biol.

[pbio-0020367-Geijtenbeek1] Geijtenbeek TB, Van Vliet SJ, Koppel EA, Sanchez-Hernandez M, Vandenbroucke-Grauls CM (2003). Mycobacteria target DC-SIGN to suppress dendritic cell function. J Exp Med.

[pbio-0020367-Glickman1] Glickman MS, Jacobs WR (2001). Microbial pathogenesis of Mycobacterium tuberculosis: Dawn of a discipline. Cell.

[pbio-0020367-Guinn1] Guinn KM, Hickey MJ, Mathur SK, Zakel KL, Grotzke JE (2004). Individual RD1-region genes are required for export of ESAT-6/CFP-10 and for virulence of Mycobacterium tuberculosis. Mol Microbiol.

[pbio-0020367-Herbomel1] Herbomel P, Thisse B, Thisse C (1999). Ontogeny and behaviour of early macrophages in the zebrafish embryo. Development.

[pbio-0020367-Hsu1] Hsu T, Hingley-Wilson SM, Chen B, Chen M, Dai AZ (2003). The primary mechanism of attenuation of bacillus Calmette-Guerin is a loss of secreted lytic function required for invasion of lung interstitial tissue. Proc Natl Acad Sci U S A.

[pbio-0020367-Kaufmann1] Kaufmann SH (2000). Is the development of a new tuberculosis vaccine possible?. Nat Med.

[pbio-0020367-Lawn1] Lawn SD, Butera ST, Shinnick TM (2002). Tuberculosis unleashed: The impact of human immunodeficiency virus infection on the host granulomatous response to Mycobacterium tuberculosis. Microbes Infect.

[pbio-0020367-Lewis1] Lewis KN, Liao R, Guinn KM, Hickey MJ, Smith S (2003). Deletion of RD1 from M. tuberculosis mimics BCG attenuation. J Inf Dis.

[pbio-0020367-Locksley1] Locksley RM, Killeen N, Lenardo MJ (2001). The TNF and TNF receptor superfamilies: Integrating mammalian biology. Cell.

[pbio-0020367-Mahairas1] Mahairas GG, Sabo PJ, Hickey MJ, Singh DC, Stover CK (1996). Molecular analysis of genetic differences between Mycobacterium bovis BCG and virulent M. bovis. J Bacteriol.

[pbio-0020367-Pallen1] Pallen MJ (2002). The ESAT-6/WXG100 superfamily—And a new gram-positive secretion system?. Trends Microbiol.

[pbio-0020367-Peters1] Peters W, Ernst JD (2003). Mechanisms of cell recruitment in the immune response to Mycobacterium tuberculosis. Microbes Infect.

[pbio-0020367-Pym1] Pym AS, Brodin P, Brosch R, Huerre M, Cole ST (2002). Loss of RD1 contributed to the attenuation of the live tuberculosis vaccines Mycobacterium bovis BCG and Mycobacterium microti. Mol Microbiol.

[pbio-0020367-Pym2] Pym AS, Brodin P, Majlessi L, Brosch R, Demangel C (2003). Recombinant BCG exporting ESAT-6 confers enhanced protection against tuberculosis. Nat Med.

[pbio-0020367-Ramakrishnan1] Ramakrishnan L, Falkow S (1994). Mycobacterium marinum persists in cultured mammalian cells in a temperature-restricted fashion. Infect Immun.

[pbio-0020367-Ramakrishnan2] Ramakrishnan L, Valdivia RH, McKerrow JH, Falkow S (1997). Mycobacterium marinum causes both long-term subclinical infection and acute disease in the leopard frog *(Rana pipiens)*. Infect Immun.

[pbio-0020367-Ramakrishnan3] Ramakrishnan L, Federspiel NA, Falkow S (2000). Granuloma-specific expression of *Mycobacterium* virulence proteins from the glycine-rich PE-PGRS family. Science.

[pbio-0020367-Sherman1] Sherman DR, Voskuil M, Schnappinger D, Liao R, Harrell MI (2001). Regulation of the Mycobacterium tuberculosis hypoxic response gene encoding α-crystallin. Proc Natl Acad Sci U S A.

[pbio-0020367-Sherman2] Sherman DR, Guinn KM, Hickey MJ, Mathur SK, Zakel KL (2004). Mycobacterium tuberculosis H37Rv: ΔRD1 is more virulent than M. bovis bacille Calmetter-Guerin in long-term murine infection. J Inf Dis.

[pbio-0020367-Smith1] Smith I (2003). Mycobacterium tuberculosis pathogenesis and molecular determinants of virulence. Clin Microbiol Rev.

[pbio-0020367-Stamm1] Stamm LM, Morisaki JH, Gao LY, Jeng RL, McDonald KL (2003). Mycobacterium marinum escapes from phagosomes and is propelled by actin-based motility. J Exp Med.

[pbio-0020367-Stanley1] Stanley SA, Raghavan S, Hwang WW, Cox JS (2003). Acute infection and macrophage subversion by Mycobacterium tuberculosis require a specialized secretion system. PNAS.

[pbio-0020367-Tailleux1] Tailleux L, Schwartz O, Herrmann JL, Pivert E, Jackson M (2003). DC-SIGN is the major Mycobacterium tuberculosis receptor on human dendritic cells. J Exp Med.

[pbio-0020367-Teitelbaum1] Teitelbaum R, Schubert W, Gunther L, Kress Y, Macaluso F (1999). The M cell as a portal of entry to the lung for the bacterial pathogen Mycobacterium tuberculosis. Immunity.

[pbio-0020367-Tekaia1] Tekaia F, Gordon SV, Garnier T, Brosch R, Barrell BG (1999). Analysis of the proteome of Mycobacterium tuberculosis in silico. Tuber Lung Dis.

